# From Hematopoietic Stem Cell Transplantation to Chimeric Antigen Receptor Therapy: Advances, Limitations and Future Perspectives

**DOI:** 10.3390/cells10112845

**Published:** 2021-10-22

**Authors:** Elisaveta Voynova, Damian Kovalovsky

**Affiliations:** Experimental Transplantation and Immunotherapy Branch, Center for Cancer Research, National Cancer Institute, National Institute of Health, Bethesda, MD 20892, USA; elisaveta.voynova@nih.gov

**Keywords:** HSCT, T-cell, CAR-T, TIL, cancer

## Abstract

Chimeric antigen receptor (CAR) T-cell therapy was envisioned as a mechanism to re-direct effector T-cells to eliminate tumor cells. CARs are composed of the variable region of an antibody that binds a native cancer antigen coupled to the signaling domain of a TCR and co-stimulatory molecules. Its success and approval by the U.S. Food and Drug Administration for the treatment of B-cell malignancies revolutionized the immunotherapy field, leading to extensive research on its possible application for other cancer types. In this review, we will focus on the evolution of CAR-T cell therapy outlining current technologies as well as major obstacles for its wide application. We will highlight achievements, the efforts to increase efficacy and to evolve into an off-the-shelf treatment, and as a possible future treatment for non-cancer related diseases.

## 1. Cell Therapy—A Little Bit of History

Cell therapy is the transplantation of healthy cells into a patient to replace damaged or dysfunctional cells. These donor cells can be autologous, derived from the patient receiving the treatment, or allogeneic, in which cells are derived from a healthy donor. Allogenic hematopoietic stem cell transplantation (HSCT), also called bone marrow transplant, consists of a stem cell graft from a healthy donor to replenish the immune system and has been in practice as cancer treatment for more than 50 years [1].

The first transfusion of human bone marrow was performed to a patient with aplastic anemia in 1939 as an attempt to raise her leukocyte and platelet counts [2]. After World War II, biomedical research was focused to develop treatments for aplasia patients caused by exposure to radiation by the atomic bomb. Studies performed in mice and guinea pigs showed that they recovered from a lethal dose of radiation after receiving bone marrow from a healthy donor [3]. However, the observation that a bone marrow transplant could be curative for cancer occurred later in 1956. Barnes and colleagues treated two groups of mice with acute leukemia. Both groups were irradiated as anti-leukemic therapy and received either a syngeneic or allogenic marrow graft to salvage from bone marrow aplasia. They observed that mice receiving syngeneic marrow died from leukemia relapse while mice receiving allogenic marrow did not experience relapse but died from a wasting syndrome [4].

The first allogeneic HSCT was pioneered by E. Donnall Thomas and his team at the Fred Hutchinson Cancer Research Center in 1957 [5]. In this study, six patients were treated with radiation and chemotherapy followed by intravenous infusion of bone marrow from a normal donor to reestablish the damaged or defective cells. However, allogenic HSCT carried high mortality due to a mismatch between donor and recipient human leukocyte antigens (HLA), which led donor immune cells to attack the host, called graft versus host disease (GvHD). Methods to identify HLA were only later developed, allowing donor and recipient HLA matching and the first transplantation of an HLA-matched unrelated donor [6]. In 1977, from 100 leukemia patients treated with chemotherapy and radiation therapy, only 13 patients were alive without disease 1 to 4.5 years after HSCT [7]. This rescue was later improved by an earlier application of allogeneic HSCT in the course of acute leukemia, leading to first remission in 50% of AML patients transplanted [8]. In 1990, E. Donnall Thomas won a Nobel Prize for his discoveries in cell transplantation. However, disease relapse and GvHD remained the two major causes of mortality in HSCT.

## 2. Adoptive Cell Therapy

Adoptive cell transfer (ACT) therapies utilize autologous immune cells, in particular, T-cells, which are isolated, may be genetically engineered, ex vivo expanded, and reinfused back into patients to eliminate cancer cells [9].

Rosenberg et al. showed that systemic administration of autologous lymphokine-activated killer cells (LAK), isolated from blood, and accompanied by recombinant IL-2 may lead to an antitumor effect in metastatic melanoma, colon cancer, renal-cell cancer and adenocarcinoma patients that failed standard therapy [10]. Although 9 out of 25 patients showed partial responses and only 1 a complete response, these results showed that immune cells able to target the tumor are present in patients and can be re-invigorated by cytokines. This approach was based on the previous demonstration that established tumors in several mouse models can start regression by the systemic administration of LAK [11,12,13]. However, a major difficulty on the application of this approach is the inability to generate sufficient numbers of autologous human cells with antitumor reactivity that could be used for systemic therapy [10]. Dr. Rosenberg and others showed that tumor-infiltrating lymphocytes (TILs) present in solid melanoma tumors could be isolated, expanded in vitro and reinfused into patients, leading to clinical responses in around 50% of melanoma patients [14,15,16].

## 3. T-Cell Receptor (TCR)-Engineered Lymphocytes

The advance in molecular biology led to the identification of tumor-associated antigens (TAAs), which are predominantly expressed in tumor cells that could be specifically targeted by single TCRs. Advances in the identification and cloning of single TCRs recognizing specific TAAs and the ability to incorporate these TCRs in T-cells via transduction with retrovirus and lentivirus, allowed the generation of TCR-engineered lymphocytes recognizing single TAAs. TCRs recognizing the melanoma antigen MART-1 and the cancer-testing antigen NY-ESO-1 were cloned. In 2006, Rosenberg and colleagues transferred TCR T-cells specifically recognizing MART-1 in 15 patients, two of whom achieved regression and still showed high levels of engineered cells in circulation 1 year after infusion [17]. This was the first clinical use of TCR T-cell therapy, showing the feasibility, safety and efficacy of introducing engineered T-cells in patients. TCR trials were conducted for non-melanoma malignancies targeting the cancer-testis antigen NY-ESO-1 [18], the onco-fetal carcinoembryonic antigen CEA for colorectal cancer [19], or melanoma antigen-encoding genes (MAGEs) [20]. Recent TCR therapy targeting HPV-16 E6 protein in cervical cancer has shown objective partial responses in 2 out of 12 patients [21].

TCR T-cell therapy can recognize a wide range of targets, including cell surface as well as intracellular proteins. Peptides derived from these proteins are presented by the major histocompatibility complex (MHC) or HLA in humans. Therefore, correct MHC/HLA expression and unaltered antigen processing are necessary for effector T-cells to recognize and kill target cells.

## 4. First Generation CARs

**Figure 1:**
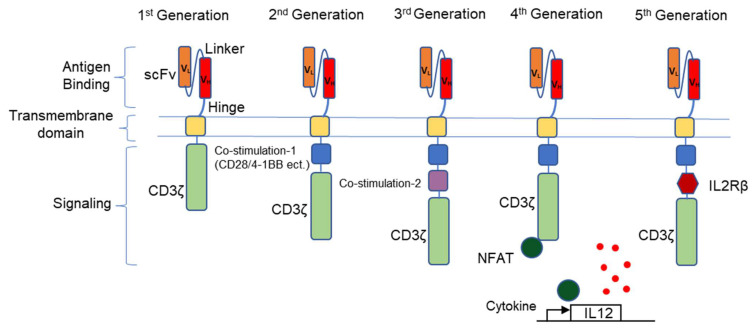
Structure of the generations of chimeric antigen receptors. The first generation of CARs contain only one intracellular signal component CD3ζ, one more costimulatory molecule is added to the second generation of CARs and another costimulatory molecule is added to the third generation of CARs. Fourth generation of CAR T-cells based on the second generation can activate a downstream transcription factor to induce cytokine production after target recognition. The fifth generation of CARs based also on the second generation incorporated an additional activation domain derived from IL2Rβ.

Tumor cells are under constant immunosurveillance by the immune system and the downregulation of leukocyte antigen (HLA) class I molecule occurs frequently in many cancers as a primary mechanism of escape. Downregulation of HLA expression was reported in 16–50% of primary tumors and 37–69% of metastasis depending on tumor type [22]. In 1989, Zelig Eshhar and colleagues postulated that if T-cells would have the ability to recognize surface tumor antigens, the recognition of tumor cells would be independent of HLA. They envisioned that replacing domains of the TCR with the variable domain of antibodies would generate a chimeric antigen receptor (CAR) with specificity towards surface proteins on tumor cells [23]. They generated a CAR comprised of the variable domain from the antibody SP6 recognizing the hapten TNP. Hybridoma cells transfected with this construct were able to activate and secrete IL-2 in response to immobilized TNP-protein conjugates and killed TNP-bearing cells across different strains and species, proving that recognition of the targeted antigen can bypass the need of antigen processing and presentation in cancer cells. They later improved this approach by generating a single-chain fragment (scFv) encoding both heavy and light variable regions joined by a linker sequence and coupled to the CD3 signaling domain, negating the need for multiple gene transfers to achieve antibody-like receptor specificity [24]. This construct represented the first-generation CAR (Figure 1).

Although some positive clinical outcomes were achieved using first generation CAR targeting disialoganglioside GD2 in pediatric patients with neuroblastoma [25,26], early clinical cancer trials in patients with renal cancer [27] and neuroblastoma [28], did not demonstrate long-term cell engraftment nor anti-tumor efficacy.

## 5. Second and Third Generation CARs

The first generation of CAR T-cells involved genetically modified T-cells with antibody specificity by expressing immunoglobulin TCR chimeric molecules as functional receptors, these CAR-T cells were unable to persist and provide long term efficacy. The physiologic recognition of tumor antigens by T-cells is mediated by the TCR–CD3 complex. However, this complex alone is insufficient to trigger productive T-cell responses, which require a second signal by the simultaneous engagement of co-stimulatory receptors. The second-generation CAR incorporated co-stimulatory molecules in its design bypassing this limitation (Figure 1). Human primary T lymphocytes retrovirally transduced with the 3G6-CD28 CAR secrete interleukin 2, survive proapoptotic culture conditions, and selectively underwent clonal expansion in the presence of an anti-idiotypic antibody specific for 3G6-CD28 [29]. In 2002, Michel Sadelain and colleagues optimized the CAR design by integrating the intracellular domains of TCR and the key co-stimulatory receptor CD28 within a single molecule to help sustain T-cell expansion, function, and in vivo persistence [30]. Other similar second-generation CARs subsequently emerged, incorporating different co-stimulatory domains, such as 4-1BB. Crystal Mackall and colleagues showed that 41BB may be superior to CD28 as it can decrease T-cell exhaustion induced by antigen-independent tonic CAR signaling, thereby improving antitumor efficacy [31]. To increase the signaling potential of CARs, third-generation CARs were designed, which combine two costimulatory domains (CD28 and 4-1BB) in their cytoplasmic domain. A high affinity CAR against mesothelin containing both CD28 and 4-1BB co-stimulatory domains led to the elimination of large tumors in a xenograft model [32]. Similarly, CD20-CAR-T cells containing both CD28 and 4-1BB molecules present increased persistence in a B-cell leukemia xenograft model [33], suggesting that combination of two co-stimulatory domains may be beneficial.

## 6. CAR T-Cell Clinical Data

In 2010, James Kochenderfer and colleagues achieved a breakthrough with a CAR-T cell therapy. They reported tumor regression in a patient with advanced follicular lymphoma, who received two infusions of autologous T-cells genetically engineered to express a CAR specifically recognizing the B-cell antigen CD19 [34]. The finding that CAR-T cells have activity in patients advanced the field of ACT, which saw dramatic progress in the following years and remarkable results in B-cell malignancies, including pediatric and adult acute lymphoblastic leukemia (ALL), chronic lymphocytic leukemia and multiple myeloma. In 2017, two studies, the phase II ZUMA-1 [35] trial led by Sattva Neelapu and a case-series study led by Carl June, validated the efficacy of CD19 CAR-T cells in patients with refractory B-cell leukemia and lymphoma. Later that year, CD19 CAR-T cells received U.S. FDA (Food and Drug Administration) approval for the treatment of children with ALL and adults with aggressive lymphomas. A phase I clinical trial of a CAR-T-cell therapy (bb2121) that targets the B cell–maturation antigen (BCMA) shows an acceptable toxicity profile and signs of antitumor activity in relapsed or refractory multiple myeloma. This trial and others indicate the clinical utility of using CAR-T cells to target antigens other than CD19 [36]. Idecabtagene vicluecel (Abecma) an anti-BCMA CAR-T-cell treatment for multiple myeloma was recently approved by the FDA. Treatment shrank tumors in 72% of patients and eliminated tumors in 28% of patients that remained in remission for a median of 11 months [37].

The U.S. FDA has approved several chimeric antigen receptors for the treatment of malignant leukemias (Table 1). Kymriah (tisagenlecleucel) is the first approved chimeric antigen receptor T-cell therapy targeting CD19, for the treatment of children and young adults up to the age of 25 years with B-cell precursor ALL. ALL is the most common hematological malignancy, as well as the most common cause of cancer-related deaths, among children in the United States. Tecartus (brexucabtagene autoleucel, formerly KTE-X19) was approved in July 2020 for the treatment of advanced mantle cell lymphoma. After that Yescarta (axicabtagene ciloleucel) was approved for the treatment of adults with diffuse large B-cell lymphoma (DLBCL) and was granted accelerated approval for the treatment of adult patients with relapsed or refractory follicular lymphoma in Feb 2021. FDA approved Breyanzi (lisocabtagene maraleucel) for the treatment of adult patients with relapsed or refractory large B-cell lymphomas. Approved CD19-CAR therapies achieved objective response rates in over 80% of patients and complete responses in over 65% of patients that have exhausted all other treatment options, a unique achievement for CAR-T-cell therapies.

## 7. Limitations and Challenges of CAR-T-Cell Therapy

### 7.1. Identification of Tumor-Specific Targets

Despite the successful treatment of hematological malignancies using CAR-T cells, no similar success was achieved for solid tumors. One of the longstanding challenges is the identification of antigens that are expressed in solid tumors but not in healthy cells. The successful treatment of hematological malignancies using CAR-T cells was achieved by targeting the B-cell lineage marker CD19. This treatment eliminates healthy as well as malignant B-cells [38]. In this case, the depletion of B-cells is not lethal, and it can be effectively managed by administration of intravenous immunoglobulins. Contrary to hematological malignancies, treatment of solid tumors cannot rely on targeting lineage markers as it would lead to the destruction of healthy tissue and significant on-target/off-tumor toxicity. For example, Her2 is an established therapeutic target for a large subset of women with breast cancer and a variety of antibodies impairing signaling is approved as breast cancer treatment [39]. However, a study reported a lethal outcome of a patient treated with CAR-T cells targeting Her2, as CAR-T cells attacked the epithelial cells of the lung which express very low levels of the antigen [40]. GD2 is an antigen highly expressed in neuroblastoma and Dinutuximab, an anti-GD2 antibody, is approved by the FDA and EMA for pediatric neuroblastoma [41]. However, a high affinity anti-GD2 CAR for neuroblastoma shows on-target/off-tumor toxicity due to low levels of GD2 expression in the brain, resulting in fatal encephalitis in a mouse model [42].

These outcomes highlight that a major limitation of CAR-T-cell therapy is to find cell surface antigens that are specifically expressed by tumor cells. Although some antigens may be predominantly expressed in cancer cells, it is always possible that very low and undetected amounts are also expressed in other cell types which may lead to severe toxicities. Although preclinical models may predict some of these toxicities, differences in antigen expression between humans and mice exist and the risk of toxicities cannot be completely ruled out.

### 7.2. Safety Switches

To safeguard from possible toxicities, strategies to eliminate “toxic” CAR-T cells were developed. One strategy is the engineering of “switches” or “suicide genes” to eliminate CAR-T cells. The inducible caspase 9 (iCasp9) consists of a fusion protein of the human caspase 9 with the drug binding domain FKBP12-F36V. When exposed to a synthetic dimerizing drug, iCasp9 becomes activated, triggering the apoptosis of cells expressing this construct. Patients with acute leukemia received an allogeneic hematopoietic stem cell followed by iCasp9-modified donor T-cells to enhance immune reconstitution, the dimerizing agent was subsequently administered to four patients who developed GvHD, resulting in elimination of >90% of the modified T-cells within 30 min and resolution of GvHD thus demonstrating the rapid depletion of CAR-T cells [43]. Another strategy involves pharmacological regulation of CAR expression by incorporation of a degradation tag into the CAR (SWIFF-CARs) [44]. In this CAR construct the degradation tag is constantly released from the CAR by a self-cleavage domain that is composed of a protease and a protease-target site. Continuous self-cleavage separates the degradation tag from the CAR leading to CAR presence on the cell surface (ON). Pharmacological inhibition of the protease by a small molecule leads to degradation of the CAR (OFF). Another general safety switch drug that could also be used for all CARs is the tyrosine kinase inhibitor dasatinib, which is already FDA-approved for the treatment of Philadelphia chromosome-positive chronic myeloid leukemia and ALL. Dasatinib inhibits T-cell activation, and it was shown to function as an immediate off-switch in CD8+ and CD4+ CAR-T cells halting cytolytic activity and cytokine production without affecting T-cell viability for several days. This effect was reversible and CAR-T-cell function was restored after discontinuation of the treatment, providing a universal safety switch to regulate CAR-T-cell function [45].

### 7.3. Antigen Downregulation or Antigen Escape

The target antigen may disappear from the tumor after therapy due to selective pressure by CAR-T cells which leads to tumor relapses. Glioblastoma, one of the most aggressive primary brain tumors, has shown antigen loss in mice after treatment with anti-IL13Rα2-CAR.IL15 T cells due to downregulated IL13Rα2 expression [46]. A clinical study demonstrated that using an anti-EGFRvIII-CAR in patients with glioblastoma, resulted in antigen loss and downregulation of the EGFR/EGFRvIII receptor appeared to promote T-cell resistance [47].

### 7.4. Antigen Heterogeneity within the Tumor

Antigen heterogeneity may also impair the success of CAR-T-cell therapy directed towards a single antigen. A CAR specific for both HER2 and MUC1 for breast cancer showed that dual targeting can lead to the delivery of complementary signals that enhance T-cell proliferation [48], and dual-target CARs specific for HER2 and IL13Rα2 mitigate antigen escape in a xenograft glioma model [49].

Despite the success of CAR T-cells in ALL, cancer relapse was observed in approximately 40% of B-ALL patients that achieved high remission rates after treatment with CD19-CAR-T cells. Interestingly, half of the relapsed patients showed a lack of expression of the CD19 target molecule. Single cell expression analysis from a B-ALL patient before and after CAR treatment identified the presence of CD19 negative B-ALL clones previous to the CAR-T-cell treatment, suggesting selective amplification of CD19 negative clones already present in the patient [50]. The high heterogeneity of the expression of most target antigens in tumors calls for the simultaneous use of multiple targets to achieve higher efficacy and to reduce the risk of relapse. Single bi-specific CAR that simultaneously target two antigens such as CD19/CD20 or CD19/CD22 were tested and showed clinical efficacy in patients with B cell malignancies [51].

### 7.5. Immunosuppression and “4th and 5th Generation CARs”

Immunosuppression within the tumor is a major challenge that blunts an effective CAR-T-cell response. The tumor microenvironment contains immunoregulatory cells, cells with an altered metabolism, as well as physical barriers that may prevent the infiltration of effector T-cells into the tumor site [52]. Direct intra-tumoral injection of CAR-T cells to bypass physical barriers was tested. In a small trial, patients with metastatic breast cancer-expressing c-Met were injected intratumorally with c-Met-CAR T-cells. Results showed that the approach was safe and well tolerated and analysis of tumors revealed extensive intratumoral necrosis [53]. Upregulation of immunosuppressive PD-L1 in tumor cells is a mechanism preventing T-cell killing. Combination immunotherapy that consists of human anti-carbonic anhydrase IX (CAIX)-targeted CAR-T cells engineered to secrete human PD-L1 antibodies at the tumor site showed effective regression of renal cell carcinoma in a humanized mouse model [54]. Another approach to overcome the intratumoral immunoregulatory circuits, to enhance the potency of CAR-T cells or increase their fitness, is to genetically modify CAR-T cells to secrete different cytokines—“fourth generation/armored CARs” or cytokine receptors “fifth generation CAR” (Figure 1).

Anti-CD19-CAR-T-cells expressing IL-12 showed greater efficacy than anti-CD19-CAR-T cells alone [55]. Pre-clinical studies suggest that targeted delivery of IL-12 into the tumor environment using CAR-T cells redirected against VEGF receptor-2 results in regression of multiple vascularized tumors [56]. Effective tumor treatment was associated with enhanced infiltration, and the expansion and persistence of anti-VEGFR2 CAR plus IL-12-engineered T-cells compared to anti-VEGFR-2 CAR T-cells alone [56]. The remarkable increase in CAR-T efficacy by IL-12 overexpression was correlated to its inhibitory effect on myeloid-derived suppressor cells within the tumor microenvironment [57,58]. However, overexpression of IL-12 in effector T-cells was associated with systemic toxicities in mouse models and transduction of an inducible IL-12 gene in tumor-infiltrating lymphocytes in a clinical study led to higher levels of toxicities, precluding its general use to overcome the immunoregulatory tumor microenvironment [59]. A few successful studies have shown that T-cell-encoded IL-15 increases the viability and proliferation of human peptide-specific T cells, in line with IL-15 function in effector T-cell survival and memory formation [60].

Recently described fifth-generation CARs incorporate a third cytokine signal in their design to increase activity. A recent study showed greater antitumor effects with minimal toxicity of a CD19 CAR also encoding the cytoplasmic domain from the interleukin 2 receptor b-chain (IL2rb) and a STAT3- binding motif compared with the same construct without IL2rb [61].

### 7.6. Cytokine Storm

Another safety concern of CAR therapy is the development of a cytokine storm which is associated with robust antitumor responses mediated by large numbers of activated CAR-T cells [38]. Uncontrolled secretion of cytokines causes side effects, such as high fever and hypotension, potentially resulting in organ failure. Effective IL-6 blockade using tocilizumab was shown to counterbalance severe clinical outcomes without compromising T-cell efficacy. It was recently shown that IL-6-knockdown in CAR-T cells reduces IL-6 secreted by monocytes and may contribute to the reduction of the cytokine storm [62]. Alternatively, the selection of CAR constructs with a lower activation profile in vitro may lead to lower toxicities in vivo. This was recently shown in a first in-human clinical trial of a novel fully human anti-CD19-CAR construct (Hu19-CD828Z). Treatment with Hu19-CD828Z led to neurotoxicities in only 5% of patients compared to 50% in patients receiving the FMC63-28Z anti-CD19-CAR construct [63].

## 8. CAR-T Cell for T-Cell Malignancies

Several groups have evaluated if CAR-T cells would be effective to target T-cell malignancies. Although positive results were observed in some mouse models, the expression of the target antigen in CAR-T cells impairs efficacy as it leads to a fratricide effect in which CAR-T cells are the target of a CAR-T cell attack [64]. Genetic deletion of the target antigen CD7 via CRISPR in conjunction with incorporation of a CD7-CAR was shown as an alternative in a T-ALL xenograft model [65]. Fraticide resistant CD3^−/−^TCR^−/−^CD7^−/−^ CD3-CAR/CD7-CAR T-cells were recently shown to eliminate primary T-ALL cells [66].

## 9. Off-the-Shelf CARs

CAR-T cell therapy is highly personalized, which requires administration of autologous cells to prevent GvHD. To achieve this, cells need to be isolated from the patient, genetically modified in the laboratory, amplified, and reinfused into the patient. This process is highly costly and confines its application as a last resort when other therapies have failed.

Ideally, universal donor effector cells that would not cause an allogenic response and GvHD could be used as an off-the-shelf CAR-T cell therapy. Several strategies to generate these effector cells are under investigation. As alloreactive T-cells do recognize many peptide-MHC complexes, alloreactivity still depends on the diversity of the T-cell population [67]. Adoptive transfer of allogenic T-cells with reduced TCR diversity such as virus-specific T-cells for virus-associated malignancies has shown low incidences of GvHD [68], suggesting that virus-specific T-cells may provide a suitable source of allogenic donor cells.

In a different approach, NK effector cells were evaluated as universal donor cells because of their ability to quickly respond and attack cancer cells. CAR-NK cells may present some advantages over CAR-T cells. As NK cells lack TCR, CAR-NK cells do not require HLA matching and can be used in allogeneic settings without causing GvHD. NK cells express activating receptors, such as NCRs, NKG2D, and DNAM-1, which may be engaged synergistically and independently from the CAR, triggering NK killing capability and potentially bypassing loss of targeted antigens as a tumor escape mechanism. NKs could also be derived from various sources such as blood, cord-blood, and differentiated in vitro from induced pluripotent stem cells (iPSC) [69]. The immortalized NK92 cell line was shown to be well tolerated in clinical trials. A phase I/II trial of 11 patients with relapsed or refractory CD19-positive cancers found that most patients responded to CD19-targeting CAR NK cells. This trial illustrates the therapeutic potential of CAR expression on non-T-cells and suggests that genetically engineered allogeneic NK cells could be used as an “off the shelf” cancer therapy [70]. However, contrary to effector T-cells, NK cells lack long-term persistence in vivo, as they are short lived and will not amplify after encounter with target cells, which may impair efficacy.

Prevention of GvHD by allogenic T-cells can also be achieved by disruption of the endogenous TCR in effector T-cells. Genetic engineering to disrupt the T-cell receptor constant alpha chain (TRAC) was studied to eliminate TCR expression in T-cells [71]. The feasibility of this approach was first tested using TALEN technology to simultaneously disrupt TRAC and CD52 to render CD19-CAR T-cells resistant to destruction by the chemotherapeutic agent anti-CD52 antibody alemtuzumab [72]. More recently, introduction of a CAR into the TRAC locus via CRISPR/Cas9 was tested to achieve the simultaneous expression of a CAR and the disruption of an endogenous TCR. Expression of this CAR follows endogenous regulation by the regulatory elements of the TCR alpha locus and was associated with reduced exhaustion and increased efficacy [73].

Given the potential clinical benefits of an off-the-shelf CAR therapy, it is a major focus of research by several biotech companies. Fate therapeutics focuses on the development of iPSC-derived CAR-NK and CAR-T cell products, as iPSC can be maintained, genetically modified, rapidly expanded and differentiated into different effector cells in vitro, they could provide an unlimited source of allogenic effector cells. Similarly, Exacis Biotherapeutics uses iPSC as an unlimited source to derive genetically modified allogenic NK and T-cells.

However, even if allogenic NK and TCR-deficient T-cells do not cause GvHD, their effectiveness may be disrupted by an endogenous immune reaction against HLA molecules in donor cells. To prevent this immune attack, iPSCs were modified to delete HLA I and II molecules and have also incorporated PD-L1, HLA-G and CD47 to control, T and NK cell-mediated responses as well as macrophage engulfment [74]. Effector T-cells were generated from these cells [75] and this technology was recently licensed to Sana Biotechnologies.

Sana Biotechnologies is also investigating the possibility to genetically modify T-cells in vivo by injection of a fusogen carrying a transgene encoding a CAR. With this technology, as cells are modified in the patient there is no risk of alloreactivity. To perform this, viral envelop proteins are modified to incorporate an antibody-binding domain that binds CD8, directing the fusogen to specifically transduce CD8+ T-cells in the patient. Results showed efficient expression of the CD19-CAR and eradication of tumor xenografts after a single injection of the fusogen [76].

An alternative approach for in vivo non-viral gene delivery to generate CAR-T cells relies on the incorporation of genetic material by endocytosis of nanoparticles containing DNA or mRNA. Transient expression of CARs in effector T-cells was recently shown after the injection of mice with poly-beta-amino-esters (PBAE) nano-vehicles containing an anti-CD8 or anti-CD3 antibody and a mRNA encoding a CAR or TCR, leading to tumor regression in several tumor models [77]. The same group had previously shown that stable T-cell modification is feasible in vitro by using PBAE-447 nanoparticles targeting CD3 and loaded with DNA encoding a CAR and the piggyBac transposase, which would mediate incorporation of the exogenous DNA to the genome of effector T-cells [78]. In addition, PBAE-nanoparticles loaded with CRISPR-CAS9 DNA were recently shown to effectively knock-out the gene of interest in vitro [79], suggesting that stable CAR expression may be achieved by injection of antibody-directed nanoparticles in patients.

## 10. CAR-T Cells for Applications beyond Cancer

The successful ability of CAR-T cells to specifically eliminate target cells in vivo based on the expression of a single cell surface antigen has inspired researchers to develop CARs for additional applications [80]. Several clinical trials using first generation CARs were conducted early in the 1990s on HIV patients. In this approach, HIV envelope proteins that would be present in infected cells were targeted by a CD4-CAR or a scFv that binds the HIV transmembrane fusion protein gp41 [81]. This concept was shown to be safe, but failed to control virus infection [81,82,83,84]. Several groups have explored targeting HIV-infected cells using similar approaches such as CARs derived from broadly neutralizing antibodies (bNAbs) that bind conserved sites within the Env protein, including the CD4-binding site, the glycoprotein 41 (gp41) membrane-proximal external region and variable region glycans [85,86,87]. Bispecific CARs were recently developed that fuse a CD4 segment to either a bNAb-based scFv or the carbohydrate recognition domain (CRD) of a human C-type lectin [88,89]. However, these approaches were mostly unsuccessful as HIV may remain dormant on infected cells which would not be recognized by the CARs. In another application, effector CAR-T cells could be directed to kill pathological immune cells in an autoimmune disease, as it was shown for the antibody-mediated autoimmune disease pemphigus vulgaris. CAR-T cells were constructed to express the autoantigen desmoglein-3 fused to signaling domains. These CAR-T cells targeted and eliminated pathogenic B-cells secreting anti-desmoglein-3 autoantibodies via the specificity of the B-cell receptor [90]. A similar concept was recently tested to target antigen-specific T-cells in the autoimmune diabetes NOD model. In this approach, the extracellular portion of the TCR subunits were replaced by the extracellular domain of MHCII with a covalently bound peptide specific for the autoimmune T-cell clones to form a 5MCAR-CTL. A surrogate coreceptor module consisting of CD80-Lck receptor was used to provide co-stimulation by binding to CD28 or CTLA-4 molecules present in targeted T-cells. This study showed that adoptively transferred 5MCAR–CTLs can control type I diabetes by targeting autoimmune CD4+ T cells in NOD mice [91]. Novel CAR approaches aim to also target other disease types. Senescent cells are aged, non-proliferating cells that alter the tissue microenvironment. Their pathological accumulation leads to tissue damage associated with chronic inflammation and fibrosis in several pathologies. Newly developed CARs are being evaluated to target senescent cells, which may provide a therapeutic approach to a diverse variety of diseases. The urokinase-type plasminogen activator receptor (uPAR) is upregulated in senescent cells and CAR-T cells targeting uPAR were recently shown to increase survival in a mouse model of adenocarcinoma and to restore homeostasis in mice with liver fibrosis [92].

In a recent work, CAR-T cells were shown to be able to revert cardiac fibrosis in a heart disease model, one of the most common causes of human morbidity and mortality. In this study, the authors developed a CAR specific for the fibroblast activation protein (FAP), which is upregulated in fibroblasts after heart injury and cardiac fibrosis in mice. Introduction of FAP-CAR-T-cells after heart injury reduced cardiac fibrosis, establishing a proof of concept that CAR-T cells could be used to treat heart disease [93].

Since first being envisioned over 80 years ago, cell therapy has evolved to be a major treatment option for cancer patients. Early successes with allogenic HSCT for hematological malignancies were associated with high levels of morbidities and mortalities. Later, the development of CAR-T cells allowed the specific targeting of defined tumor antigens, but many challenges still remain, such as dentification of suitable tumor antigens that are not expressed in healthy tissue to prevent toxicities, the heterogeneous expression and downregulation of non-lineage markers that may lead to relapses, and the immunoregulatory microenvironment of solid tumors which render CAR-T-cell therapy ineffective. CAR-T-cell therapy is a highly costly personalized therapy. Several different technologies are currently being tested for this technology to evolve into an off-the-shelf product, which would allow its wider application. Finally, the use of CAR-T cells to treat non-cancer related diseases is a promising and emergent field. Future advances to identify specific targets and to generate an off-the-shelf CAR product would allow the field to further expand beyond its current limitations.

## Figures and Tables

**Table 1:** FDA-approved CAR-T-cell therapies.

Name	Target	Malignancy
Kymriah^TM^ (tisagenlecleucel)	CD19	Relapsed or Refractory B-cell Acute Lymphoblastic Leukemia (ALL) ^1^
Yescarta^TM^ (axicabtageneciloleucel)	CD19	Relapsed or Refractory Large B-Cell Lymphoma or Follicular Lymphoma ^1^
Tecartus^TM^ (brexucabtagene autoleucel, formerly KTE-X19)	CD19	Relapsed or Refractory Mantle Cell Lymphoma ^1^
Breyanzi^®^ (lisocabtagene maraleucel)	CD19	Relapsed or Refractory Large B-Cell Lymphoma ^1^
Abecma^®^ (idecabtagene vicleucel)	BCMA	Relapsed or Refractory Multiple Myeloma ^1^

^1^ Approved cellular and gene therapy products, FDA (https://www.fda.gov/vaccines-blood-biologics/cellular-gene-therapy-products, accessed on 10 October 2021).

## Data Availability

Not applicable.
